# Superparamagnetic colloids in viscous fluids

**DOI:** 10.1038/s41598-017-07917-y

**Published:** 2017-08-10

**Authors:** A. Darras, E. Opsomer, N. Vandewalle, G. Lumay

**Affiliations:** 1 GRASP, CESAM - Physics Department, University of Liège, B-4000 Liège, Belgium; 2 F.R.S.-FRNS, B-1000 Bruxelles, Belgium; 3 Experimental Physics, Saarland University, D-66123 Saarbrücken, Germany; 4 Université Paris Diderot, Sorbonne Paris Cité, MSC, CNRS (UMR 7057), F-75013 Paris, France

## Abstract

The influence of a magnetic field on the aggregation process of superparamagnetic colloids has been well known on short time for a few decades. However, the influence of important parameters, such as viscosity of the liquid, has received only little attention. Moreover, the equilibrium state reached after a long time is still challenging on some aspects. Indeed, recent experimental measurements show deviations from pure analytical models in extreme conditions. Furthermore, current simulations would require several years of computing time to reach equilibrium state under those conditions. In the present paper, we show how viscosity influences the characteristic time of the aggregation process, with experimental measurements in agreement with previous theories on transient behaviour. Afterwards, we performed numerical simulations on equivalent systems with lower viscosities. Below a critical value of viscosity, a transition to a new aggregation regime is observed and analysed. We noticed this result can be used to reduce the numerical simulation time from several orders of magnitude, without modifying the intrinsic physical behaviour of the particles. However, it also implies that, for high magnetic fields, granular gases could have a very different behaviour from colloidal liquids.

## Introduction

Superparamagnetic colloids are magnetic nanoparticles inserted in a matrix of non-magnetic material (polystyrene or silica) to obtain particles with diameter *d* ranging from 100 nm to a few micrometres. These composite particles are combining a quasi-zero remanent magnetisation and a high magnetic response^1–3^. In applications, the superparamagnetic colloids are functionalised to capture specific targets such as protein, cell or bacteria^4–7^. After the capture, an inhomogeneous external magnetic field is applied to separate the superparamagnetic particles by magnetophoresis^8^. Moreover, the formation of chains along the magnetic field enhances the separation process. This technique is used for protein isolation, cell separation, waste capture, bacteria processing, chromatography, etc.^1, 4–7, 9–17^. More complex structures of superparamagnetic colloids can be obtained by using rotating fields, even possibly leading to microswimmers or tracers of local dynamics^18–32^. Those complex structures open ways to new kinds of applications as they have unique optical properties and offer tunable structures able to adapt to their environment and execute functional tasks^20, 23, 26^. However, the previous studies about those complex structures focus on the properties of the structures obtained, without having a deep understanding of their formation process. To our knowledge, the only system for which some model of growth has been published in the literature up to now is the colloidal chains formed under constant magnetic fields.

In colloidal science, it is well known that particles tend to agglomerate due to van der Waals interactions^33, 34^. In the present experiments, this agglomeration is prevented by covering the particles with carboxyl charged groups. These charged groups create a short range repulsion between the particles, typically within a range of 10 nm between the particles^2, 35^. This ensures the stability of the dispersion. In the following, this electrostatic interaction is considered to define an effective size of the particles for the contact of particles which is 10 nm wider than the natural size of the particles^1^. However, when an external magnetic field $\overrightarrow{B}$ is applied on the suspension, the superparamagnetic particles acquire a magnetic dipole $\overrightarrow{\mu }=\chi V\overrightarrow{B}$, with the magnetic susceptibility *χ* of the particles and their volume $V=\frac{4}{3}\pi {R}^{3}$, given their radius *R*. The particles then interact with each other through dipolar interactions. The potential energy of magnetic interaction between two identical particles at distance *r* is therefore given by

1 $$U(r,\theta )=\frac{{\chi }^{2}4\pi {R}^{6}{B}^{2}}{9{\mu }_{0}}(\frac{1-3\,{\cos }^{2}\,\theta }{{r}^{3}}),$$

with *θ* being the angle between the magnetic field $\overrightarrow{B}$ and the line joining the centre of the particles. The force associated with this interaction is then

2 $${\overrightarrow{F}}_{m}=-\overrightarrow{\nabla }U(r,\theta )=\frac{3{\mu }_{0}{\mu }^{2}}{4\pi {r}^{4}}((1-3\,{\cos }^{2}\,\theta ){\overrightarrow{e}}_{r}-\,\sin \,2\theta {\overrightarrow{e}}_{\theta }),$$

with ${\overrightarrow{e}}_{r}=\frac{\overrightarrow{r}}{r}$ and ${\overrightarrow{e}}_{\theta }={\overrightarrow{e}}_{z}\times {\overrightarrow{e}}_{r}$ if ${\overrightarrow{e}}_{z}$ is the unitary vector perpendicular to the plan containing ${\overrightarrow{e}}_{r}$ and $\overrightarrow{B}$ (if $\overrightarrow{B}$ and ${\overrightarrow{e}}_{r}$ are parallel, the orientation of ${\overrightarrow{e}}_{\theta }$ is meaningless since *θ* = 0). Two particles then attract each other when they are aligned with the field $\overrightarrow{B}$, while they repel each other if they are side-by-side. This interaction implies that two particles tend to aggregate in a chain aligned with the magnetic field $\overrightarrow{B}$. Several studies, both experimental and theoretical, have shown that superparamagnetic colloids self-organise into chains under those conditions, through diffusion-limited aggregation^13, 36–41^. Moreover, this aggregation is reversible, meaning that the chains break up if the magnetic field $\overrightarrow{B}$ is suppressed^28, 42^. Experimentally, chains of several particles are typically observed^36, 37, 39^ and the growth is successfully described on short time (typically up to 300 s) by a Smoluchowsky equation, predicting a power law behaviour of the mean size of the chains 〈*s*〉 ∝ *t* ^*z*^ after a transient behaviour^36–40^. Current research usually focuses on more complex structures that have recently been observed under those conditions^43, 44^ and new theoretical models are currently studied in order to take them into account and describe their properties^41, 45, 46^.

However, only little attention has been given to the influence of the viscosity of the surrounding fluid. Yet since the aggregation is diffusion-limited, the viscosity of the fluid could modify the aggregation mechanism through its influence on the diffusion coefficient. Moreover, the equilibrium state reached after a long time is still challenging on some aspects. Indeed, recent experimental measurements show deviations from pure analytical models in extreme conditions^12, 43, 44^. Current techniques of numerical simulations would require several years of computing time to reach equilibrium state under some conditions: actual experiments last for hours and one second of simulation currently takes from 300 to 1100 hours of computer time. The most challenging situations are the ones leading to long chains, for which experiments and analytical models disagree^12, 43^.

In the present work, we first provide experimental observation of the influence of viscosity on the formation of such chains, and compare our results with previous theoretical models. Then, a modification of this viscosity is tested in simulations, in order to explore a wider range of viscosity. This allows to test conditions which are closer to dust suspensions in the air, where we observe another aggregation regime. Moreover, a nice application would be to use it to speed up the simulation time of colloidal system.

## Experimental Setup

**Figure 1:**
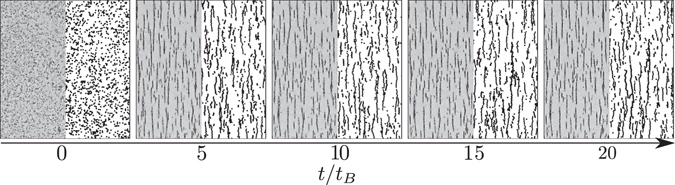
Evolution of the chain formation along scaled time as observed from one of our experiments (left side of each picture) and one of our simulations (right side of each picture). The pictures are part of images obtained with a magnetic field *B* = 12 G. One can observe the formation of chains aligned with the external magnetic field and a qualitative similarity between experiments and simulations. The characteristic time *t*_*B*_ is defined in equation (3).

A sketch of the experimental pictures is presented in Fig. 1. The experiments were performed with superparamagnetic microspheres dispersed in glycerol-water mixtures (Estapor^®^ M1-070/60), with a volumic fraction of *ϕ* = 2 10^−3^. The viscosity of each liquid phase was measured before adding the particles to the suspension with a Haake-MARS rheometre. The measurements were consistent with the available tables^47^. The range of viscosity we used goes from 1 mPa s to 100 mPa s. We measured, by image analysis, a radius of particles *r* = 0.6 ± 0.3 *μ*m while the mean susceptibility, obtained by magnetophoresis^2, 48–50^, was *χ* = 0.09 ± 0.03. Those values are consistent with previous characterisation of that sample found in the literature^28, 29^. The suspension was placed inside a cylindrical chamber of diameter *D* = 5 mm and thickness *h* = 50 *μ*m. The chamber was formed by two parallel glass plates. The first glass plate was covered with a 50 *μ*m layer of epoxy with the exemption of a circular region. A suspension droplet of 1 *μ*l was placed inside this region. Afterwards, the second glass plate was placed on the first one. A small quantity of low-viscosity silicon oil was placed on the epoxy to assess the watertightness of the chamber. A constant and homogeneous magnetic field *B* was generated by a constant current in surrounding coils at the beginning of each experiment. The magnetic field produced by those coils was characterised with a Hall probe and was homogeneous within the precision range of the probe of 2% around the cell. The current in the coils had a constant intensity controlled by a programmable DC power supply GenH-750W from TDK Lambda, with a precision of 0.01 A. The suspension was observed from the bottom with a 10x magnification. The microscope used was an inverted microscope Olympus IX73, connected to a 4070M-CL Thorlabs Camera with 2048 by 2048 pixels of 16 Bits depth. The images were recorded with a frame rate of 1fps.

## Experimental Results

**Figure 2:**
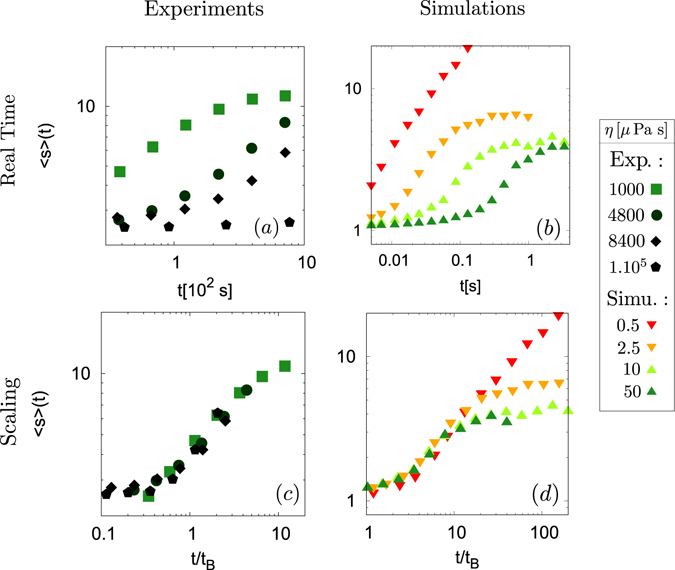
Log-log plots of the evolution of the mean size of the chains 〈*s*〉 during experiments ((**a**) and (**c**)) and simulations ((**b**) and (**d**)). The mean chains length is expressed in mean diameter of particles. After a transient behaviour whose duration depends on the viscosity, a power law growth is obtained with an exponent close to 0.5. In (**a**) and (**b**), a clear trend can be seen on that graph: the higher the viscosity, the slower the growth. In (**c**) and (**d**), the mean size of the chains is plotted as a function of the parameter *t*/*t*_*B*_, where the characteristic time is defined in equation (3). All experimental curves then collapse. In simulations, if the magnetic field *B* = 12 G, curves collapse only for *η* > *η*_*c*_, with ${\eta }_{c}\in ]2.5\,\mu {\rm{Pa}}\,{\rm{s}}\mathrm{;\; 5}\,\mu {\rm{Pa}}\,{\rm{s}}[$. Colours and points shapes correspondence are described in the box on the right side.

The time evolution of the system is shown in Fig. 2(a). We measured, as a function of time, the normalised mean size 〈*s*〉 of the chains, expressed in particles diameter, formed by the colloidal particles when the magnetic field is applied with an intensity of about 12 G. This size is obtained through image analysis, by averaging the major axis of ellipses fitted on each chain in the image (at least 2000 chains). For short time experiments, after a transient behaviour, we obtained a power law growth, with an exponent *z* close to 0.5, as observed in previous studies^36, 37, 39^. In Fig. 2(a), a clear trend can be seen on that graph: the higher the viscosity, the slower the growth.

For long times and low viscosity experiments, a saturation of the mean size 〈*s*〉 is observed as expected from the theoretical development of Andreu *et al*.^12^ and observed in some of our previous experiments (see Fig. 2(a))^44^.

Those differences of behaviour can be explained on the basis of the Smoluchowsky equation and the mechanism of diffusion-limited aggregation. Indeed, a diffusion-limited aggregation, taking into account an effective capture volume as suggested by Fermigier *et al*., has a characteristic time scale ${t}_{B}=\frac{{R}^{2}}{24[{\mathrm{(1/3)}}^{\mathrm{1/2}}-{\mathrm{(1/3)}}^{\mathrm{3/2}}]D{\rm{\Gamma }}\varphi }$ ^36^, where *R* is the radius of the particles, *D* is the diffusion coefficient of a single particle, ${\rm{\Gamma }}=\frac{{\chi }^{2}\pi {R}^{3}{B}^{2}}{9{\mu }_{0}{k}_{B}T}\equiv \frac{2{U}_{0}}{{k}_{B}T}$ is a dimensionless parameter comparing the magnetic energy with the thermal energy *k*_*B*_ *T* (*χ* is the magnetic susceptibility of the particles and *B* is the magnetic field amplitude) and *ϕ* is the volume fraction of the particles. By using the Stokes-Einstein relation $D=\frac{{k}_{B}T}{6\pi R\eta }$, where *η* is the viscosity of the fluid, we can then write this characteristic time as

3 $${t}_{B}=\frac{6\pi \eta {R}^{3}}{48[{\mathrm{(1/3)}}^{\mathrm{1/2}}-{\mathrm{(1/3)}}^{\mathrm{3/2}}]{U}_{0}\varphi }\mathrm{.}$$

When the data are expressed as a function of this dimensionless time $\frac{t}{{t}_{B}}$, we obtain the plot in Fig. 2(c), where all the curves collapse. Since the only difference between our experiments is the viscosity *η* of the fluid, this collapse highlights that, in the range of our experiments, the viscosity simply slows down the aggregation process, without modifying further the intrinsic physical mechanism of aggregation.

But one can wonder: if increasing the viscosity simply slows down the growth process of the chains, would reducing the viscosity speed up this growth? This is what we tried to achieve in some numerical simulations, since fluids with lower viscosity than water are not abundant. Actually, the range of viscosity we explored is closer to the viscosity of the air. This means that experiments corresponding to those conditions should be performed with micrometric superparamagnetic dust or powder suspended in a gas, which is not easily available.

## Numerical Simulations: Methods

Numerical simulations are useful tools to compare ideal experiments with, on the one hand, actual experiments or, on the other hand, theoretical expectations. Comparing with actual experiments can indicate if all the key physical ingredients are taken into account in the models. It can also be used to test some models in range of parameters which are not accessible experimentally. In the case of the analytical models for the mean chains length at saturation^12, 51^, such simulations can (dis)confirm the mathematical approximations. However, each second of numerical simulation can require between 300 and 1100 hours of computer time, the most challenging situations being the ones leading to long chains^12, 43^. Speeding up the simulations is then critical to study the cases corresponding to experiments which can last up to five hours.

In our study, simulations are realised using a Soft Sphere Discrete Element Method^52–54^ taking into account the dipole-dipole interactions between the colloidal particles as well as the Brownian agitation in the system. The algorithm progresses with a constant time step Δ*t* and solves Newton’s equations of motion at each iteration. The different force models we considered are described here below.

The normal contact force acting on two impacting particles is modelled using a linear spring-dashpot. The repulsive component is proportional to the overlap *δ* between particles while the energy dissipation during the collision is taken into consideration via an additional damping force. Altogether one obtains,

4 $${\overrightarrow{F}}_{n}=-{k}_{n}\delta \overrightarrow{n}-\gamma \frac{d\delta }{dt}\overrightarrow{n},$$

where *k*_*n*_ is the spring stiffness, $\overrightarrow{n}$ the normal unit vector and *γ* a complex function of *k*_*n*_ and the restitution coefficient *ε*. The tangential contact force is proportional to the relative slipping velocities *v*_*s*_ of the particles. Moreover, it is bounded by Coulomb’s criterion which yields in,

5 $${\overrightarrow{F}}_{t}=-{k}_{t}{v}_{s}\overrightarrow{t},\quad |{F}_{t}|\le {\mu }_{d}|{F}_{n}|,$$

where *k*_*t*_ is a large positive constant, $\overrightarrow{t}$ the tangential unit vector and *μ*_*d*_ is the dynamic friction coefficient.

When exposed to an external magnetic field, the colloidal particles acquire a magnetic dipole inducing long-range interactions between them. The associated force ${\overrightarrow{F}}_{m}$, given in equation (2), can directly be used in the simulations for each pair of particles. However, in order to gain some computational time, we introduced a cut-off distance of about 12*r* by using a linked-cell method^55^.

The random motion of a particle due to its interaction with surrounding fluid molecules in the heat bath can be described by using a Langevin equation^56^. The drag force is considered to be ${\overrightarrow{F}}_{d}=-6\pi R\eta \overrightarrow{v}$, where $\overrightarrow{v}$ is the velocity of the particle. The Brownian force ${\overrightarrow{F}}_{b}$ is modelled as a Gaussian white noise process^57, 58^. One has,

6 $${\overrightarrow{F}}_{b}=\overrightarrow{\xi }6\pi R\eta \sqrt{\frac{2D}{{\rm{\Delta }}t}}$$

where Δ*t* is the time step in the simulation and $\overrightarrow{\xi }$ is a vector of three random gaussian variables with zero mean and unit variance.

Since sedimentation plays an important role in the dynamics of our system, gravity and buoyancy, noted respectively ${\overrightarrow{F}}_{g}$ and ${\overrightarrow{F}}_{a}$, have to be included.

It is worthwhile to notice that changing the viscosity parameter *η* then modifies both the drag force ${\overrightarrow{F}}_{d}$ and the Brownian force ${\overrightarrow{F}}_{b}$ in the simulations (indeed, *F*_*d*_ ∝ *η* and ${F}_{b}\propto \eta \sqrt{D}\propto \sqrt{\eta }$). Besides this, all the other parameters remain constant since they depend only on temperature and particles’ properties.

## Numerical Simulations: Results

Simulations allow to test the scaling resulting from the former relation in equation (3) relating the characteristic time of the system *t*_*B*_ ∝ *η* to the viscosity *η* for low viscosities. If this scaling is valid for every value of viscosity, it can be used to speed up the simulations related to our experiments. Indeed, to some extent, it would mean simulations performed with a fluid viscosity which is lower than the ones available experimentally are faster but the colloidal assemblies retain the same geometrical properties and aggregation mechanisms. We then performed simulations for viscosities varying from *η* = 5 10^−7^ Pa s to 10^−4^ Pa s. As illustrated in curve Fig. 2b, the same trend as in experimental is observed: the higher the viscosity, the slower the growth. In curve and Fig. 2d, the curves efficiently collapse with the scaling *t*/*t*_*B*_, except if the viscosity *η* is smaller than a critical value *η*_*c*_, with ${\eta }_{c}\in ]2.5\,\mu {\rm{Pa}}\,{\rm{s}}\mathrm{;5}\,\mu {\rm{Pa}}\,{\rm{s}}[$ in the case of Fig. 2.

One might wonder if this viscosity threshold arises from a numerical bias. But in our numerical model, the main approximation depending on viscosity is the use of the Brownian force ${\overrightarrow{F}}_{b}$ to create the diffusive motion of the particles, according to the Langevin model^56^. This model is valid as long as the characteristic autocorrelation time of the Brownian force, which is determined by the time step Δ*t* ≈ 10^−7^ s in our simulations, is negligible compared to the characteristic viscous time $\frac{2\rho {R}^{2}}{9\eta }\approx \frac{{10}^{-10}\,{\rm{Pa}}\,{{\rm{s}}}^{{\rm{2}}}}{\eta }$, with $\rho =1200\frac{{\rm{kg}}}{{{\rm{m}}}^{{\rm{3}}}}$ the density of the particles. This viscous time is defined as the characteristic speed relaxation time of a particle with a non-zero initial speed, if only the drag force is acting on it. We then have to respect ${10}^{-7}{\rm{s}}\ll \frac{{10}^{-10}\,{\rm{Pa}}\,{{\rm{s}}}^{{\rm{2}}}}{\eta }\iff \eta \ll {10}^{-3}\,{\rm{Pa}}\,{\rm{s}}$, which is always the case in our simulations since the maximum value of the viscosity we used is *η* = 10^−4^ Pa s. Actually, we chose it accordingly to the time step fixed by the contact dynamics ${\rm{\Delta }}t\ll 2\pi \sqrt{\frac{m}{{k}_{n}}}$ ^52–54^. To be certain of the validity of this assumption, we also performed some simulations with a reduced time step of Δ*t* ≈ 10^−8^ s but no modification of the simulations’ results was then observed, for any of the viscosity we used. Eventually, this shows that the Langevin model is only better when the viscosity decreases, and the Langevin model can not be responsible for an artificial onset of the behaviour transition we observed in the simulations.

This means that no numerical effect is responsible for this behaviour transition. Actually, we believe that a new physical aggregation mechanism appears when the viscosity is below a critical threshold. Our hypothesis is that this threshold comes from the rise of a non-negligible drift due to the magnetic interactions of the grains. Indeed, every particle close to another one has a drift velocity due to the magnetic force exerted by this neighbour. On long times $t\gg {t}_{\eta }$ greater than the viscous time ${t}_{\eta }=\frac{m}{6\pi R\eta }$, since the particles are in a viscous fluid, the motion equation for average values of a single particle can be written $\langle {\overrightarrow{F}}_{m}\rangle =6\pi R\eta \langle {\overrightarrow{v}}_{D}\rangle$, where $\langle {\overrightarrow{v}}_{D}\rangle$ is then the mean drift velocity of the particles. The mean magnetic force $\langle {\overrightarrow{F}}_{m}\rangle$ depends on the actual particles density in the neighbourhood of the considered sphere, as well as its distance from the closest neighbour. However, a characteristic value is the maximum of this force ${F}_{m,M}=\frac{\pi {\chi }^{2}{R}^{2}{B}^{2}}{6{\mu }_{0}}$, which is then related with the maximum drift velocity as

7 $$\langle {v}_{D,m}\rangle =\frac{{F}_{m,M}}{6\pi R\eta }=\frac{R{\chi }^{2}{B}^{2}}{36{\mu }_{0}\eta }\mathrm{.}$$

The impact of the magnetic interaction in the motion of the particles can then be assessed by the ratio of this maximum drift speed 〈*v*_*D*,*m*_〉 and the characteristic root mean square speed due to Brownian agitation ${\tilde{v}}_{th}=\sqrt{\frac{3{k}_{B}T}{m}}$. This defines a kind of Peclet number, comparing the drift transport due to magnetic interaction to the stochastic transport due to the Brownian motion

8 $$Pe\equiv \frac{\langle {v}_{D,m}\rangle }{{\tilde{v}}_{th}}=\frac{{F}_{m,M}}{6\pi R\eta {\tilde{v}}_{th}}=\frac{{F}_{m,M}}{{F}_{\eta ,{\tilde{v}}_{th}}}=\frac{R{\chi }^{2}}{36{\mu }_{0}}\sqrt{\frac{m}{3{k}_{B}T}}\frac{{B}^{2}}{\eta },$$

where ${F}_{\eta ,{\tilde{v}}_{th}}$ is the modulus of drag force applied to a particle whose velocity is ${\tilde{v}}_{th}$. The third expression above then expresses that this Peclet number *Pe* is also the ratio between the maximum magnetic force and the characteristic drag force during the Brownian motion. Actually, it is easy to show that this is the only force competition which is able to explain the breakdown of scaling depending on both magnetic field and viscosity. Indeed, the only forces concerned by (at least) one of those quantities are the drag force ${\overrightarrow{F}}_{d}$, the Brownian force ${\overrightarrow{F}}_{b}$ (both depending only on viscosity) and the magnetic force ${\overrightarrow{F}}_{m}$ (depending only on magnetic field). In our simulations, the ratio $\frac{{F}_{m}}{{F}_{b}}$ is ranging from 0 to approximately 10^−2^, which indicates that the magnetic force is always negligible compared to the thermal agitation. Then this competition is not likely to give rise to any transition. However, in our simulations, the ratio $\frac{{F}_{m}}{{F}_{d}}$ ranges from 0 to approximately 4, indicating that the magnetic force becomes, in some experiments only, greater than the characteristic viscous force. Any comparison between two forces including any another force would fail to completely explain our observations by missing at least one of this parameter. This is then enough to conclude that the competition between drag and magnetic interaction is the only one relevant to explain the scaling breakdown.

**Figure 3:**
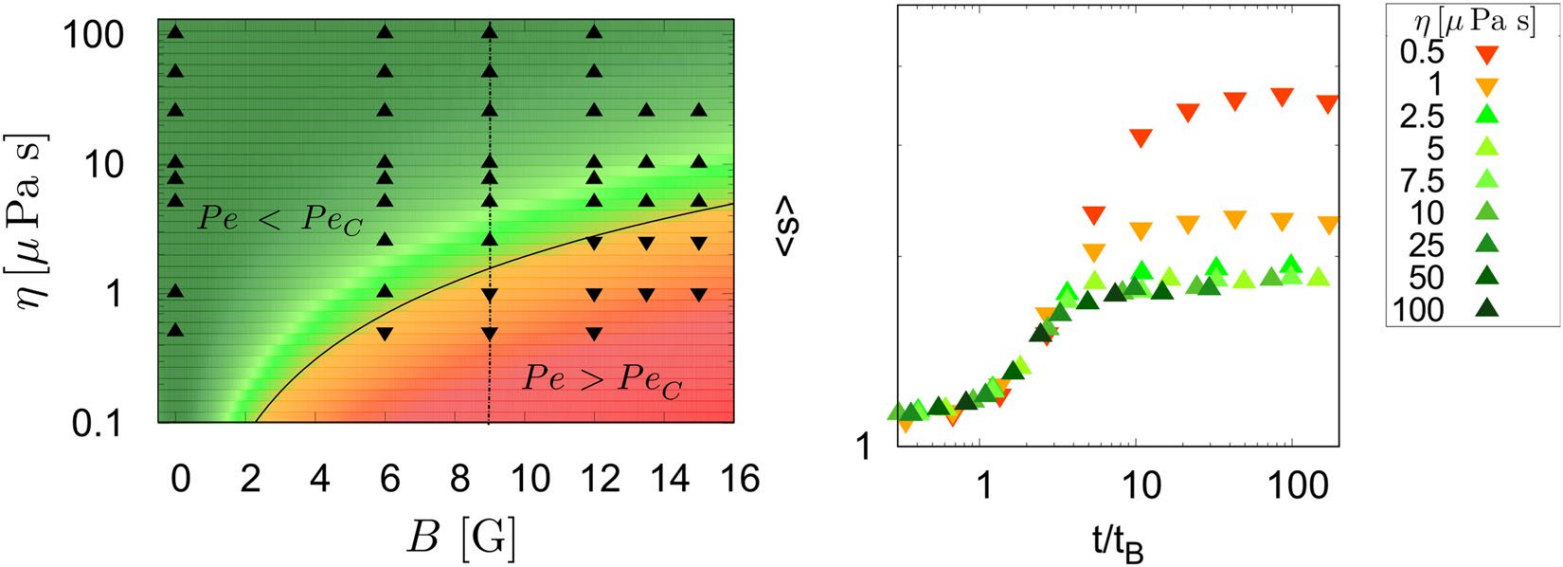
Left: global efficiency of the time scaling for all simulations. Upwards triangles are the simulations whose curves collapse with others with the same magnetic field. Downwards triangles are the simulations which don’t collapse. The black curve represents the transition *Pe* = *Pe*_*C*_, and the colour gradient is related to the difference of local Peclet number and its critical value *Pe* − *Pe*_*C*_. Right: a graphic showing the curves obtained for the simulations performed with a magnetic field *B* = 9 G. Those curves then correspond to the points on the dot-dashed line in the left graphic. The red and orange curves are not collapsing with the others and correspond to cases *Pe* > *Pe*_*C*_.

The last side of the equalities in equation (8) shows that, if the transition occurs for a critical value of *Pe*_*c*_, there is a value of viscosity *η* under which the mechanism of aggregation is intrinsically different from usual experimental observations. This minimal viscosity ${\eta }_{lim}=\frac{R{\chi }^{2}}{36{\mu }_{0}}\sqrt{\frac{m}{3{k}_{B}T}}\frac{{B}^{2}}{P{e}_{C}}$ depends on *B* ^2^ for a given set of particles. Through all the simulations we performed, with magnetic fields ranging from 0 G to 15 G, the range of viscosity for which the scaling efficiently collapses the curves is consistent with a critical value of the Peclet number *Pe*_*C*_ = 0.825 ± 0.025, see Fig. 3.

**Figure 4:**
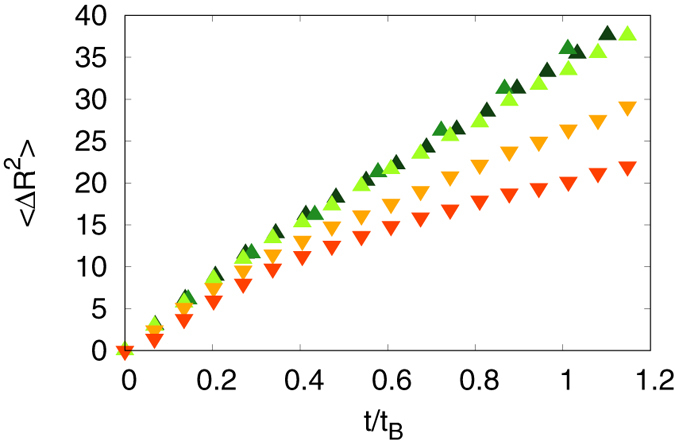
Mean Square displacement of the particles in the horizontal plane as a function of dimensionless time. The mean square displacement is expressed in particles diameter and the time has been scaled by the aggregation characteristic time *t*_*B*_ (equation (3)) to compare curves from various simulations under a magnetic field of 9 G. All the curves corresponding to points in the region *Pe* < *Pe*_*C*_ collapse, while curves in the region *Pe* > *Pe*_*C*_ have a different behaviour.

Another argument supporting this assumption is given by the analysis of the Mean Square Displacement (MSD) of the particles along time (see Fig. 4). Indeed, for a given magnetic field, the MSDs of the particles in that plane are similar for all viscosities were *Pe* < *Pe*_*C*_, while it is not if *Pe* > *Pe*_*C*_. This clearly means that when *Pe* > *Pe*_*C*_ another kinematic process, acting against the diffusion, occurs. The existence of such threshold also determines the limit of how the simulations related to a given experiment can be artificially sped up by decreasing the viscosity.

## Conclusions

Our experiments show that modifying the viscosity of the fluid only rescales the characteristic time of the agglomeration process, without modifying the underlying physical mechanisms. Then, numerical simulations showed that another aggregation regime occurs for low viscosities or high magnetic field. This indicates that, for high magnetic fields, the granular gases could have a very different behaviour from colloidal liquids. This also implies that numerical simulations can be performed on systems with lower viscosities and still be an efficient model for the experiments. The benefit of using lower viscosities is that the computer time of the simulations can be reduced from several months to a few days. This then also opens new prospects to efficiently simulate complex colloidal systems.
